# Vascular wall injury and inflammation are key pathogenic mechanisms responsible for early testicular degeneration during acute besnoitiosis in bulls

**DOI:** 10.1186/s13071-020-3959-9

**Published:** 2020-03-02

**Authors:** David González-Barrio, Carlos Diezma-Díaz, Enrique Tabanera, Elena Aguado-Criado, Manuel Pizarro, Marta González-Huecas, Ignacio Ferre, Alejandro Jiménez-Meléndez, Fernando Criado, Daniel Gutiérrez-Expósito, Luis Miguel Ortega-Mora, Gema Álvarez-García

**Affiliations:** 1 SALUVET, Animal Health Department, Faculty of Veterinary Sciences, Complutense University of Madrid, Ciudad Universitaria s/n, 28040 Madrid, Spain; 2 Department of Animal Medicine and Surgery, Faculty of Veterinary Sciences, Complutense University of Madrid, Ciudad Universitaria s/n, 28040 Madrid, Spain; 3 Livestock Health and Production Institute (ULE-CSIC), León, Spain

**Keywords:** *Besnoitia besnoiti*, Bull, Acute besnoitiosis, Testicular degeneration, Lesions

## Abstract

**Background:**

Bovine besnoitiosis, caused by the apicomplexan parasite *Besnoitia besnoiti*, is a chronic and debilitating cattle disease that notably impairs fertility. Acutely infected bulls may develop respiratory signs and orchitis, and sterility has been reported in chronic infections. However, the pathogenesis of acute disease and its impact on reproductive function remain unknown.

**Methods:**

Herein, we studied the microscopic lesions as well as parasite presence and load in the testis (pampiniform plexus, testicular parenchyma and scrotal skin) of seven bulls with an acute *B. besnoiti* infection. Acute infection was confirmed by serological techniques (IgM seropositive results and IgG seronegative results) and subsequent parasite detection by PCR and histological techniques.

**Results:**

The most parasitized tissue was the scrotal skin. Moreover, the presence of tachyzoites, as shown by immunohistochemistry, was associated with vasculitis, and three bulls had already developed juvenile tissue cysts. In all animals, severe endothelial injury was evidenced by marked congestion, thrombosis, necrotizing vasculitis and angiogenesis, among others, in the pampiniform plexus, testicular parenchyma and scrotal skin. Vascular lesions coexisted with lesions characteristic of a chronic infection in the majority of bulls: hyperkeratosis, acanthosis and a marked diffuse fibroplasia in the dermis of the scrotum. An intense inflammatory infiltrate was also observed in the testicular parenchyma accompanied by different degrees of germline atrophy in the seminiferous tubules with the disappearance of various strata of germ cells in four bulls.

**Conclusions:**

This study confirmed that severe acute besnoitiosis leads to early sterility that might be permanent, which is supported by the severe lesions observed. Consequently, we hypothesized that testicular degeneration might be a consequence of (i) thermoregulation failure induced by vascular lesions in pampiniform plexus and scrotal skin lesions; (ii) severe vascular wall injury induced by the inflammatory response in the testis; and (iii) blood-testis barrier damage and alteration of spermatogenesis by immunoresponse.
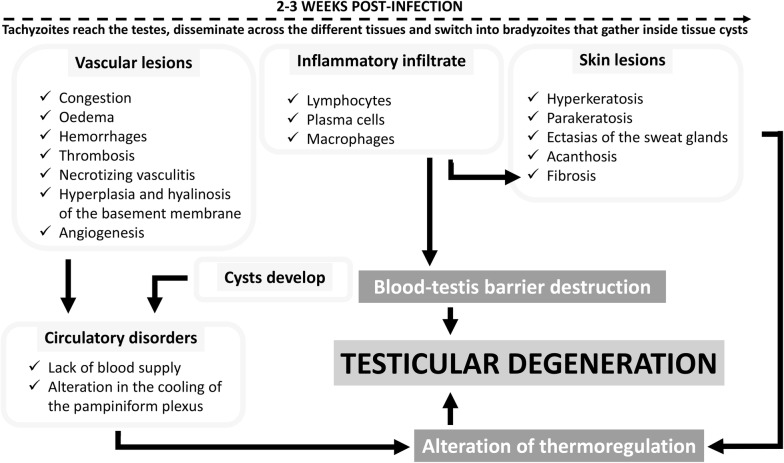

## Background

Bovine besnoitiosis is a cattle parasitic disease caused by the cyst-forming apicomplexan protozoan *Besnoitia besnoiti* [1]. The disease has traditionally been endemic in sub-Saharan Africa and Asia. However, the European Food Safety Authority [2] alerted to the re-emergence of this disease in Europe in areas where the disease was traditionally endemic to neighbouring countries (the Alentejo region in Portugal, the north-eastern part of Spain and French Pyrenees) [3]. Besnoitiosis is a chronic and debilitating disease that primarily affects beef cattle; apart from a low body score, systemic clinical signs and skin lesions, reproductive failure is the major concern since males may develop infertility, sterility or even die during the acute phase [4, 5].

Several authors have reported that beef cattle are more susceptible to the infection than dairy cattle that could be attributed to risk factors associated with the management husbandry system. In fact, in beef cattle herds males appeared to show acute clinical signs more frequently and higher mortality rates as they might be more exposed than females due to natural mating [6, 7]. Recently, Gazzonis et al. [8] reported that males presented a greater risk of infection, with an incidence of infection of 60% vs 38.8% in females.

Clinical besnoitiosis outcome occurs in two sequential phases. First, an acute febrile phase appears due to the fast-replicating tachyzoite stage in endothelial and mononuclear cells. This phase is characterized by generalized oedema and ocular and nasal discharge [4]. Bulls may develop orchitis, and alveolar and interstitial oedemas appear in the lungs, causing respiratory disorders. Severely affected animals may die due to respiratory dysfunction and nephrotic syndrome [9]. It has been suggested that tachyzoite replication in endothelial cells may cause vascular damage since infected animals present vasculitis and thrombosis in small-to-medium diameter vessels [9, 10]. This acute phase is short-lived and rarely diagnosed [4]. The disease progression from the acute phase to the chronic phase may occur in less than one month [9, 11]. Tissue cysts develop as soon as 11 days post-infection, and their size progressively increases up to 400 µm [12]. During chronic infection, fully mature parasitic cysts can be detected in skin, scleral conjunctivae, vestibulum vaginae and non-intestinal mucosa [13]. Only a few studies have described lesions in testes and assessed seminal quality in chronically infected animals [14–16]. Infected bulls may present testicular atrophy with azoospermia. Indeed, numerous cysts have been observed in the testes, epididymis, and ampullae and in the walls of blood vessels in the pampiniform plexus that could interfere with normal spermatogenesis [9, 14, 16].

In the affected herds, most animals remain subclinically infected and only a small number develop noticeable clinical signs compatible with either acute or chronic besnoitiosis that represent the tip of the iceberg [3, 4]. Chronic besnoitiosis is easily diagnosed by the clinical detection of pathognomonic sclera tissue cysts, thickening of the scrotal skin and testicular atrophy. However, acute infection usually goes unnoticed, as the clinical signs are non-specific. Moreover, the progression of the disease may occur quickly, and severely affected animals may die due to cardio-respiratory failure before two weeks post-infection when animals have not yet developed specific IgG antibodies. In fact, only one report of acute besnoitiosis focused on histopathological findings was described in a naturally infected bull [9]. However, the pathogenic mechanisms that govern acute disease together with their impact on reproductive function remain unravelled.

The objective of this work was to determine the microscopic lesions along with their impact on reproduction function and their association with parasite stage and load in the testicles of bulls with an acute *B. besnoiti* infection. In addition, the pathogenic mechanisms that govern acute disease were also discussed

## Methods

### Samples from *B. besnoiti* naturally infected breeding bulls

Seven naturally infected breeding bulls from extensive beef herds were included in this study. The breeds of these bulls were Charolais (*n* = 4), Limousin (*n* = 1) and breed unknown (*n* = 2) (Table 1).

**Table 1:** Bulls analyzed in this study

Bull	Age	Breed	Geographical origin	Clinical signs and macroscopic lesions	Serology					
					IgM (ELISA)		IgG (ELISA)			
					RIPC %	+/−	RIPC %	+/−	WB	Avidity
1	24 months	Charolais	Madrid	Fever (41 °C), orchitis, hydrocele, edema in posterior limb, congestive scleral conjunctiva	119.2	+	80	+	+	8
2	Adult^a^	Charolais	Madrid	Respiratory problems, edema in the area of the scapula, ascites	na	na	na	na	na	na
3	Adult	Limousin	Cáceres	Orchitis, hydrocele, edema in declining areas and scleral conjunctiva	na	na	na	na	na	na
4	Adult	Unknown	Madrid	Orchitis, hydrocele, petechiae and hemorrhages in testes	137.5	+	1.0	−	−	na
5	Adult	Unknown	La Rioja	Hydrocele, congestion, adhesions	127.2	+	2.7	−	−	na
6	132 months	Charolais	Madrid	Fever (41 °C), orchitis, hydrocele, petechiae and hemorrhages in testes	167	+	4.9	−	−	na
7	35 months	Charolais	Madrid	Fever (40 °C), orchitis, hydrocele, petechiae	74.4	+	6.8	−	−	na

^a^Bull in reproductive age more than 15 months

*Abbreviations*: +. positive animal; –, negative animal; ELISA, enzyme-linked immunosorbent assay; WB, western blot; na, not available; RIPC, relative index percent

*Notes*: Bulls 2, 3 and 5 died in farm

Animals showed clinical signs or macroscopic lesions compatible with acute besnoitiosis, mainly fever and orchitis (Table 1). Three breeding bulls were found dead in the herd, and four additional bulls were sacrificed at the slaughterhouse due to the severity of clinical signs.

Blood and testes of each bull were collected, and macroscopic lesions in the testicles were recorded (Table 1). The samples were preserved at 4 °C until arrival to the laboratory. Then, blood was centrifuged at 3000×*g* for 10 min, and serum was preserved at − 20 °C. Bulls 2 and 3 died on the farms and blood was not collected. Two tissue samples were collected from each bull; one sample was frozen at − 80 °C for DNA extraction and the other was stored in 10% buffered formaldehyde for histopathological analysis.

To check the health status of sampled animals, specific antibodies against relevant cattle pathogens such as *Neospora caninum* and bovine herpesvirus-1 were investigated by ELISA techniques (an in-house tachyzoite soluble antigen based ELISA [17] and IDEXX IBR gB X3 Ab Test, (IDEXX Inc., Maine, USA), respectively). In addition, the presence of bovine viral diarrhoea virus (BVDV) was investigated by antigen detection in sera (IDEXX BVDV Ag/Serum Plus Test, IDEXX Inc.).

### Parasites and antigen production

Tachyzoites of the *in vitro* Bb-Spain 1 isolate of *B. besnoiti* [18] were purified in cold sterile PBS at pH 7.2 using disposable PD-10 desalting columns (GE Healthcare, Chalfont St. Giles, UK) and pelleted by centrifugation at 1350×*g* for 10 min at 4 °C. The pellet with tachyzoites was frozen at − 80 °C and was used as an antigen source for serological assays and for parasite DNA extraction in PCR reactions. For IgM and IgG BbSALUVET ELISA 2.0, tachyzoites were lyophilised in a Virtis Benchtop K lyophilizer. Vials for lyophilisation were prepared with 5 × 10^7^ tachyzoites per vial and resuspended in 4 ml of PBS.

### Detection of specific antibodies against *B. besnoiti* by serological analyses

#### IgM ELISA

An IgM BbSALUVET ELISA 2.0 was employed following a previously described procedure [19]. This technique was based on the employment of lyophilized *B. besnoiti* tachyzoites and the procedure described by García-Lunar et al. [20] to detect specific anti-*B. besnoiti* IgG. Herein, an incubation step with an anti-bovine IgM conjugated with horseradish peroxidase (Bovine IgM Antibody, A10-100P; Bethyl, Mongomery, USA) diluted 1:10,000 in phosphate-buffered saline containing 0.05% Tween 20 (PBST) was included. Control sera used in the ELISA came from an experimental infection carried out in calves. The positive control serum was composed of three sera from calves that were intravenously inoculated with 10^8^ tachyzoites of Bb-Spain 3 isolate and collected at 4 days post-infection (pi). The negative control serum comprised a pool of three sera from the negative control group [21]. The optical density was converted into the RIPC (relative index percent) using the formula described by García-Lunar et al. [20]. Animals with a RIPC ≥ 67.23 were considered positive.

#### IgG ELISA

Sera were analysed by BbSALUVET ELISA 2.0 [20] to discriminate between IgG seropositive and seronegative animals. A peroxidase-conjugated monoclonal goat anti-bovine IgG (Thermo Fisher Scientific, Massachusetts, USA) diluted 1:10,000 in PBST was used as a secondary antibody. The optical density was converted into RIPC using the formula described by García-Lunar et al. [20]. Animals with a RIPC ≥ 17.34 were considered positive.

#### Avidity ELISA

To discriminate between acute and chronic infection, an avidity ELISA was performed when specific IgGs were detected by BbSALUVET ELISA 2.0. The test was carried out as previously described by Diezma-Díaz et al. [22]. Sera were tested using a duplicate 4-fold dilution series starting from 1:100 to 1:102,400. After incubation with sera, an additional incubation step with 6 M urea was included for one dilution series, or with PBS-Tween for the other dilution series. The avidity index (AI) was calculated according to Aguado-Martínez et al. [23], and the cut-off to discriminate between low and high avidity was established at 50.8 according to Schares et al. [24].

#### Western blot analysis

SALUVET tachyzoite-based western blot (WB) was performed under non-reducing conditions in 12.5% polyacrylamide gels [25] to confirm the BbSALUVET ELISA 2.0 results [20]. Three main antigenic reactivity areas were described: area I (72.5, 58.9 and 51.4 kDa), area II (38.7, 31.8 and 28.5 kDa) and area III (23.6, 19.1, 17.4 and 14.5 kDa). The recognition of at least three bands in at least two of the three described areas was considered an IgG-positive result [25].

### Parasite DNA detection by conventional (PCR) and quantitative real-time PCR (qPCR)

DNA extraction of the different tissues collected (testicular parenchyma, pampiniform plexus and scrotal skin) was carried out using the Maxwell® 16 Instrument (Promega, Wisconsin, USA) with the Maxwell® 16 Tissue DNA Purification Kit (Promega) [26]. The DNA from each sample was quantified by spectrophotometry (NanoDrop, Thermo Fisher Scientific; Abs 260/280 nm ratio) and adjusted to 40 ng/μl.

*Besnoitia* spp. DNA was detected by ITS1 rDNA PCR [27]. The cycling conditions were 2 min at 95 °C, followed by 45 cycles of denaturation at 94 °C for 30 s, annealing at 58 °C for 30 s and extension at 72 °C for 1 min, followed by a final 15 min extension step at 72 °C and maintenance at 4 °C at the completion of the profile. The forward primer ITS1F (5′-TGA CAT TTA ATA ACA ATC AAC CCT T-3′) and the reverse primer ITS1R (5′-GGT TTG TAT TAA CCA ATC CGT GA-3′) were added at a concentration of 10 μM, and the remaining reagents were incorporated in the mixture, as indicated by Frey et al. [26].

The amplified products were visualized after electrophoresis on a 1.5% agarose gel containing 0.1 μl/ml GelRed™ Nucleic Acid Gel Stain (Biotium, Hayward, USA). DNA extraction and PCR were performed in separate laboratories under biosafety level II conditions (BIO II A Cabinet; Telstar, Madrid, Spain) to avoid cross contamination. DNA extracted from *in vitro* cultured *B. besnoiti* Bb-Spain 1 tachyzoites and PCR grade water were used as the positive and negative controls, respectively.

The qPCR assay for the quantification of *Besnoitia* spp. DNA was performed according to Cortes et al. [27] and Frey et al. [26]. The forward primer Bb3 (5′-CAA CAA GAG CAT CGC CTT C-3′) and the reverse primer Bb 6 (5′-ATT AAC CAA TCC GTG ATA GCA G-3′) were added at a concentration of 20 μM, and the remaining reagents were incorporated into the mixture, as indicated by Frey et al. [26]. In each PCR, 10-fold serial dilutions of genomic DNA corresponding to 0.1–10,000 Bb-Spain 1 tachyzoites were included. To quantify the amount of DNA, dilutions of DNA extracted from the liver of a cow corresponding to 100, 20, 4 and 1 ng/μl were included. The cycling conditions were 10 min at 95 °C, followed by 40 cycles of 95 °C for 15 s and 60 °C for 1 min. Fluorescence emissions were measured during the 60 °C step. A dissociation stage was added. The cycle quantification values (Cq-values) obtained for positive samples were also expressed as tachyzoites per reaction using the standard curve that was included in each run as indicated by Frey et al. [26]. Only *Besnoitia* PCR-positive samples were further analysed by qPCR.

### Histopathology

Routine paraffin wax embedding procedures were used. Tissue samples from testicular parenchyma, pampiniform plexus and scrotal skin, were fixed in 10% buffered formaldehyde, dehydrated in a graded ethanol series, and cleared in xylene. Samples were embedded in paraffin wax and sections of 5 μm thickness were cut using a sliding microtome (Leica Microsystems, Wetzlar, Germany). Sections were stained with haematoxylin and eosin (H&E), periodic acid-Schifff (PAS) and Masson trichrome (for better visualization of connective tissue) and examined by light microscopy. Photomicrographs of each studied specimen were subjected to computer-assisted image analysis using a computer coupled to an optical Olympus BX50 microscope equipped with a Colour View IIIu digital Olympus DP27 camera (Olympus, Tokyo, Japan).

Parasite cysts found in the histological sections were observed in 10 randomized fields at a magnification of 100 (10 × 10) to obtain an average number of parasite cysts and to measure the diameter of the cysts.

### Immunohistochemistry

The immunohistochemical labelling of the parasites was performed using deparaffinised tissue sections from bulls with tissue cysts: bull 2 with numerous juvenile cysts; and bull 6 with scarce juvenile cysts and severe vascular lesions in the absence of cysts in the pampiniform venous plexus. Primary in-house rabbit polyclonal antibodies against *B. besnoiti* bradyzoites [28] were used at a 1:3000 dilution following the protocol described by Frey et al. [26].

## Results

### Serological results

A total of five bulls out of seven were analysed by different serological techniques. Four bulls showed high IgM values in the absence of specific IgG (Table 1). The remaining animal presented high levels of both IgM and IgG antibodies. Thus, in this case, the avidity ELISA test was subsequently performed, and a low AI was obtained (AI = 8; Table 1).

### Parasite DNA detection

Conventional PCR results showed that *Besnoitia* spp. DNA was present in all scrotal skin samples (*n* = 7), followed by testicular parenchyma (*n* = 6) and the pampiniform plexus (*n* = 4). In total, 17 out of 21 samples analysed were positive (71%) (Table 2). In three bulls, *Besnoitia* spp. DNA was detected in the scrotal skin, testicular parenchyma and pampiniform plexus, whereas in the remaining bulls, *Besnoitia* spp. DNA was detected in at least two tissues, scrotal skin and testicular parenchyma or scrotal skin and pampiniform plexus. *Besnoitia* spp. DNA was quantified in 17 PCR-positive tissues [29], with a low parasite load in twelve of them, and the remaining five samples were negative for *Besnoitia* spp. The highest parasite load was found in scrotal skin, followed by testicular parenchyma and pampiniform plexus (Table 2).

**Table 2:** *Besnoitia* spp. detection by means of PCR and histological techniques in tissues from testicles of acutely infected bulls

Bull	Pampiniform plexus			Testicular parenchyma			Scrotal skin		
	PCR	qPCR (zoites/mg tissue)	Histology (mean cyst diameter, µm)	PCR	qPCR (zoites/mg tissue)	Histology (mean cyst diameter, µm)	PCR	qPCR (zoites/mg tissue)	Histology (mean cyst diameter, µm)
1	POS	2.0 × 10^−5^	–	NEG	nd	–	POS	NEG	50.0
2	NEG	nd	97.1	POS	3.0 × 10^−3^	20.0	POS	3.8 × 10^−4^	90.7
3	POS	3.0 × 10^−5^	–	POS	2.7 × 10^−1^	–	POS	3.3 × 10^−1^	–
4	POS	NEG	–	POS	3.0 × 10^−3^	–	POS	2.2 × 10^−1^	–
5	POS	NEG	–	POS	1.7 × 10^−1^	–	POS	2.0 × 10^−4^	–
6	NEG	nd	–	POS	3.0 × 10^−3^	–	POS	1.314	25.0
7	NEG	nd	–	POS	NEG	–	POS	NEG	–

*Abbreviations*: POS, detection of *Besnoitia* spp. DNA; NEG, absence *Besnoitia* spp. DNA; nd, not determined; –, cysts not found

### Histopathological findings

#### Gross lesions

**Fig. 1:**
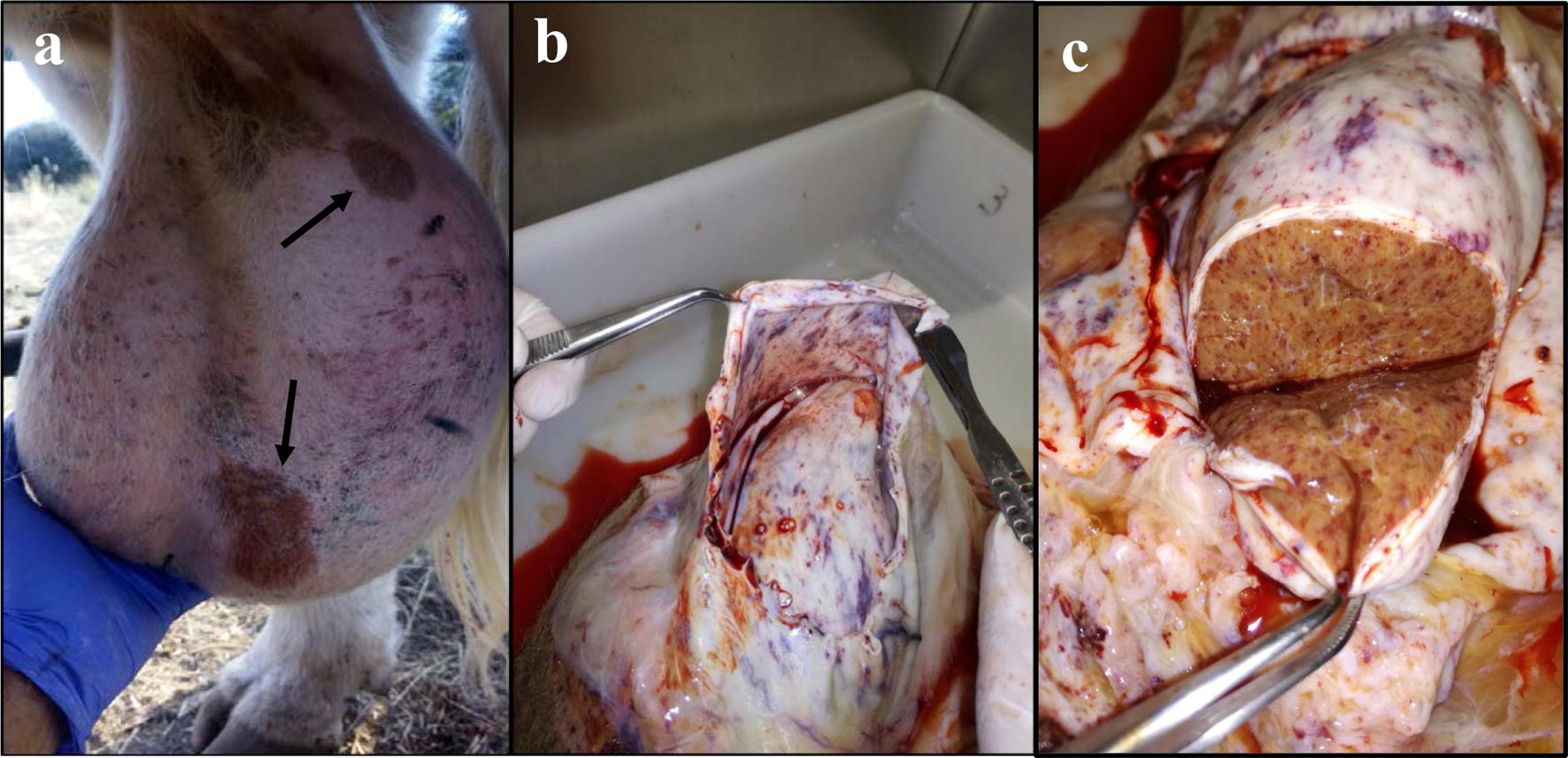
Macroscopic lesions detected in testes from acutely infected bulls. Orchitis, the black arrows indicate ulcers in scrotal skin (**a**). Hydrocele, petechiae, congestive testicular parenchyma (**b**) and increased thickness of the tunica albuginea and abnormal colour of the testicular parenchyma (**c**)

Macroscopic lesions detected in the sampled bulls are shown in Table 1 and Fig. 1. Hydrocele was the most frequent lesion along with orchitis. Other lesions, such as petechiae and haemorrhages in the testes, oedema in the limbs and congestive scleral conjunctiva, were detected in several bulls. Increased thickness of the tunica albuginea and abnormal yellowish colour of the testicular parenchyma were observed in bull 6 (Fig. 1c).

#### Microscopical findings

##### Tissue cysts

**Fig. 2:**
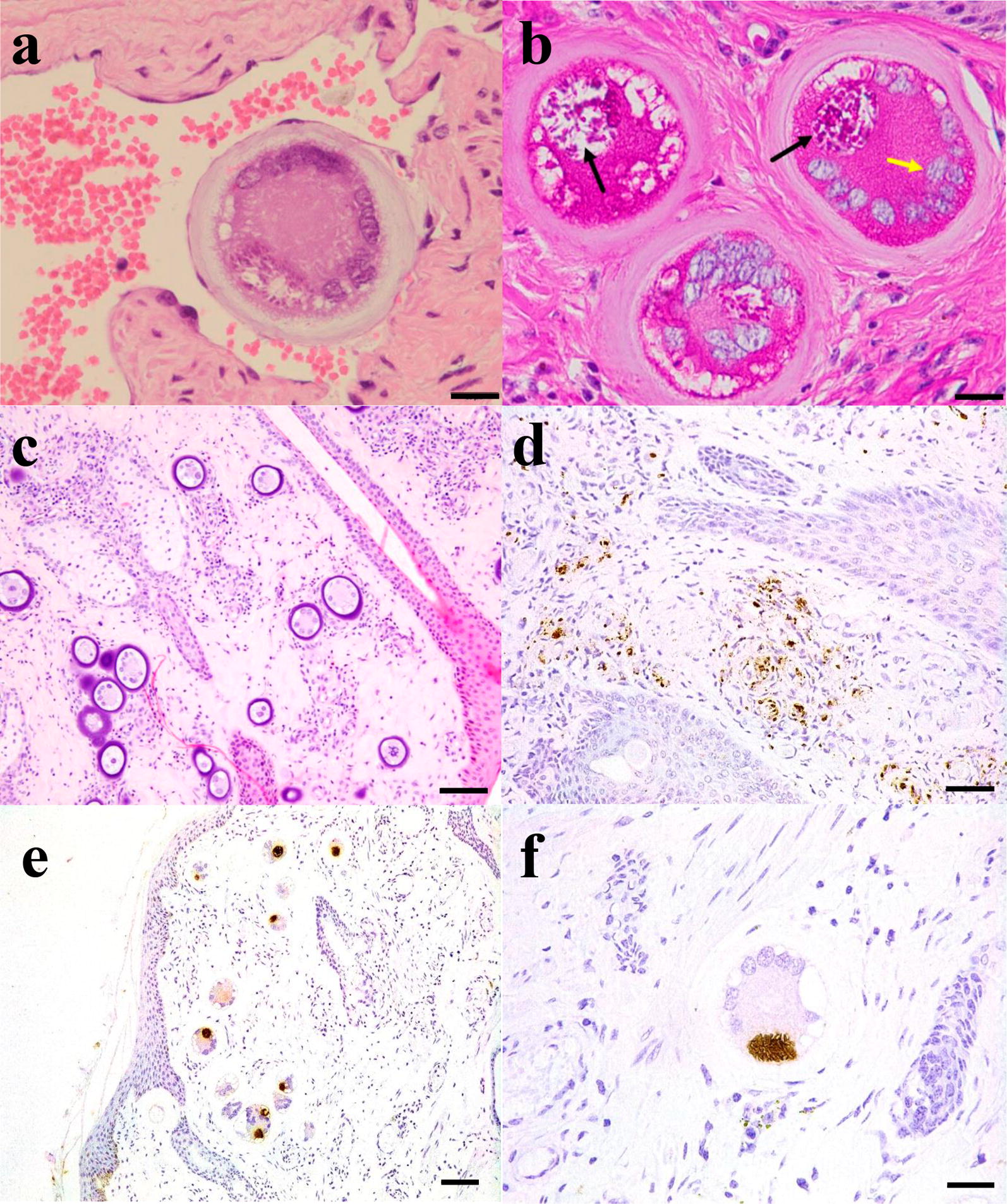
*Besnoitia besnoiti* tissue cysts in scrotal skin, testicular parenchyma and pampiniform plexus. **a**–**c** Histopathology: H&E staining in which cyst is occluding a vessel in testicular parenchyma from bull 2 (**a** ×400); histological section of scrotal skin stained with PAS staining in which tissue cysts of *B. besnoiti* are observed in the papillary layer of the dermis (**b**, **c** ×100). The black arrows indicate the bradyzoites and the yellow arrow the nuclei of the host cell. **d**–**f** Immunohistochemistry: staining of tissue section from bull 6 with rabbit-α-*B. besnoiti* showed the presence of particulate antigen of the parasite associated with vasculitis (amorphous granular debris positively labelled) in scrotal skin (**d** ×200). All tissue samples from bull 2 presented cysts with irregular distribution (**e**, **f**), the number of cysts being greater in the scrotal skin (**e**) than in the testicular parenchyma and pampiniform plexus (**f**). *Scale-bars*: **a**, **b**, **f** 20 µm; **c**, **e** 100 µm; **d** 50 µm

Juvenile tissue cysts (20–97 μm) were detected in analysed tissues from three out of seven bulls (bull 1, 2 and 6) (Table 2). These juvenile tissue cysts were found in all tissues analysed (more than 20 tissue cysts per section in scrotal skin, 2–3 tissue cysts per section in pampiniform plexus and testicular parenchyma) from bull 2 (Fig. 2a, b). The average tissue cyst diameter was 97.2, 90.68 and 20.0 μm in the pampiniform plexus, scrotal skin and testicular parenchyma, respectively. In bulls 1 and 6 (Fig. 2c), tissue cysts of 25–50 μm were found in scrotal skin. Small tissue cysts of bull 2 occurred with minimal or absent inflammatory cell reactions (Fig. 2e, f), whereas in bull 6, the presence of the parasite was detected in scrotal skin as particulate antigen (Fig. 2d) and in testicular parenchyma as parasitophorous vacuoles always associated with vasculitis (Fig. 2d). Scrotal skin cysts were mainly present in the papillary layer. Scattered tissue cysts were also found in the tunica dartos, tunica vaginalis and tunica albuginea, and occasional cysts were detected in the vessel lumen (Fig. 2a). The cysts had a homogeneous, acellular and slightly basophilic external capsule. Their content included between 5 and 20 rounded cell nuclei and a small number of bradyzoites. In PAS-stained sections, the host cell cytoplasm was grey-red in colour and sometimes stained brightly PAS-positive. The inner cyst wall stained violet-red, and bradyzoites were grey-violet with several small PAS-positive granules within the cytoplasm (Fig. 2b).

The presence of the parasite was evidenced by immunohistochemistry in all tissues of both studied bulls (bull 2 and 6), with the exception of the pampiniform plexus of bull 6.

##### Lesions

**Fig. 3:**
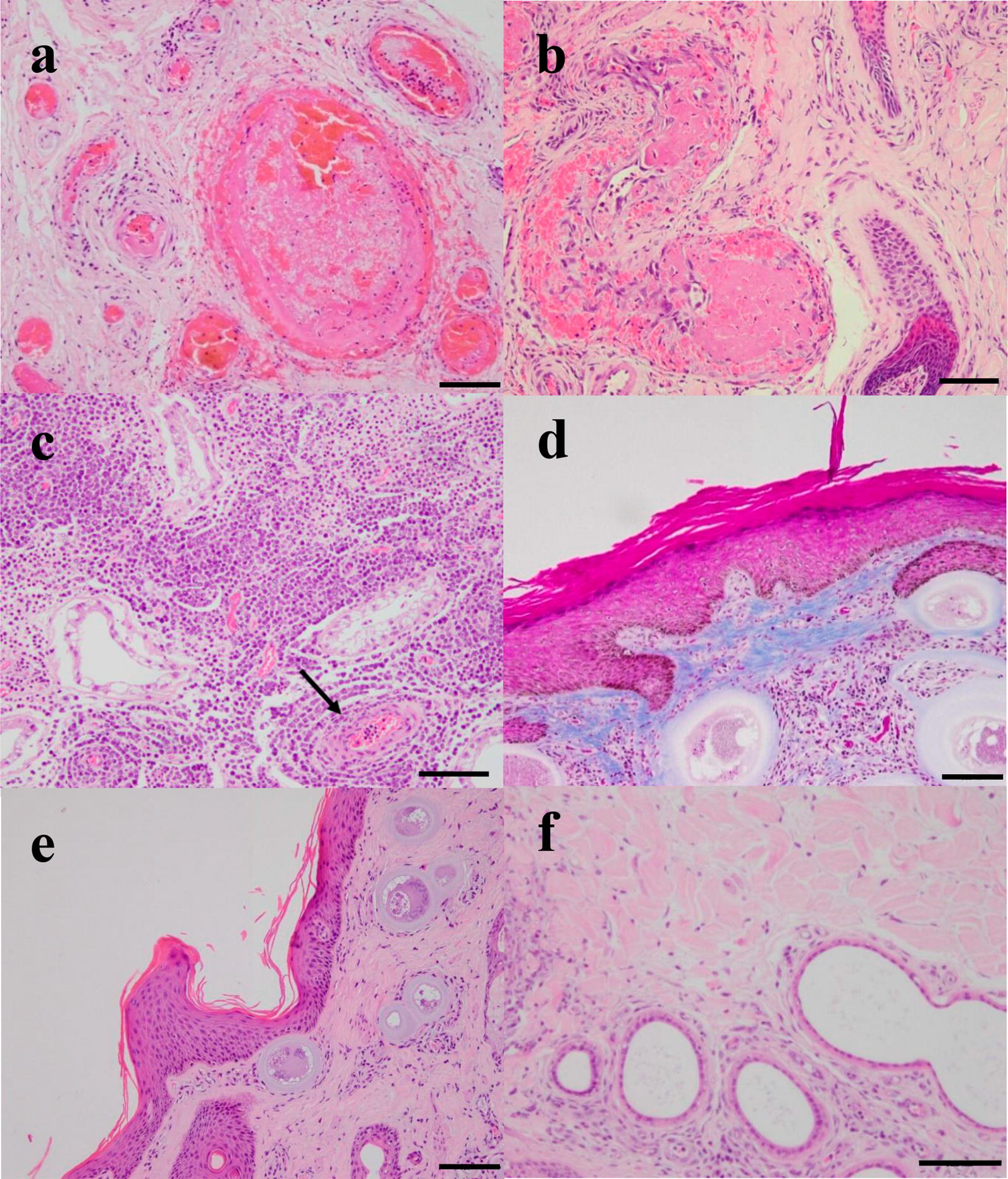
Vascular lesions and inflammation visualized in scrotal skin, testicular parenchyma and pampiniform plexus. H&E staining of the pampiniform plexus (**a**), scrotum skin (**b**) and testicular parenchyma (**c**). Thrombos (**a**, **b**), edema and necrotizing vasculitis (**b**), lymphoplasmacytic inflammation (moderate diffuse lymphoplasmacytic sclerosing type orchitis) and neovascularization marked with a black arrow (**c**). Lesions on scrotal skin with Massonʼs stain (**d**) and H&E (**e**, **f**). Thickening of the stratum corneum and the spinous layer, in the papillary layer some tissue cysts can be seen together with fibrosis (**d**), and ectasia of the sweat glands (**f**). *Scale-bars*: 100 µm

**Fig. 4:**
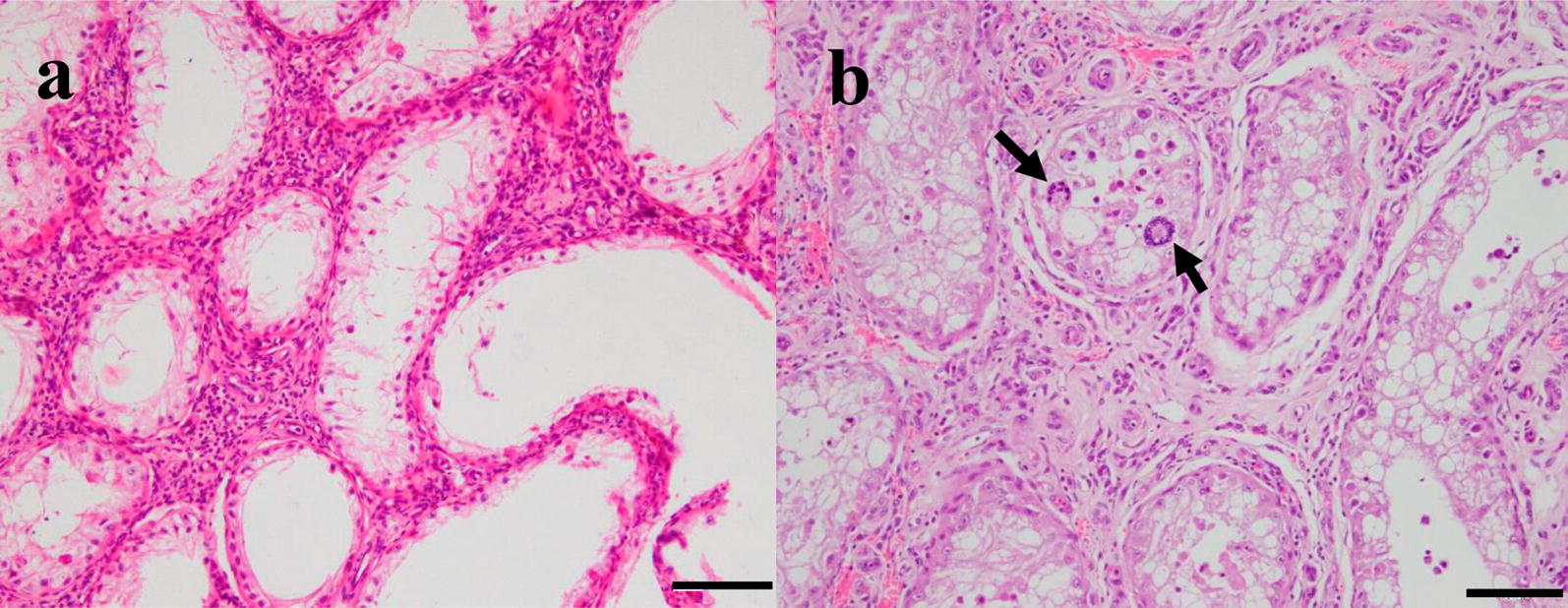
Lesions observed in the testicular parenchyma from acutely infected bulls. Testicular parenchyma with H&E staining. **a** Degenerated seminiferous tubules and absence of sperm. Seminiferous tubules with different degrees of atrophy inside which aberrant spermatids can be observed (black arrows). Surrounding the tubules there is fibrosis and inflammation (**b**). *Scale-bars*: 100 µm

Similar lesions were present in all tissues that included vascular lesions (Fig. 3a–c, in all bulls), inflammatory infiltrate (Fig. 3d, in all bulls), lesions on scrotal skin (Fig. 3d–f, in bull 1, 2, 4 and 7), fibroplasia (Fig. 3e, in bull 1, 2, 4 and 7) and aspermia (Fig. 4a, in bull 1, 2, 4 and 6). Vascular injury predominated in all tissues and was marked in the pampiniform plexus and scrotal skin from all bulls. In particular, thrombosis, oedema, necrotizing vasculitis, angiogenesis, and marked congestion and haemorrhages were observed in the blood vessels of the scrotal skin, tunica albuginea, testicular parenchyma and pampiniform plexus (Fig. 3a–c).

In bulls 1, 2, 4 and 7, diffuse thickening of the stratum corneum (hyperkeratosis) was observed in the epidermis; in one case (bull 2), nuclei were present in the keratinized cells (parakeratotic). An increase in the spiny layer (acanthosis) was also observed in bulls 1, 2, 4, and 7. We found areas with loss of epithelium (ulcers) and pustules in bull 6. In all cases, marked ecstasies of the sweat glands were evident (Fig. 3f).

Inflammatory infiltrate was predominant in all tissues. Some vessels had lymphocytic infiltrates in the wall (vasculitis). Hyalinosis and thickening of the basement membrane was also observed. Likewise, moderate hyperplasia of the smooth muscle fibres of the middle layer of both arterioles and venules was observed (Fig. 3d, e, bull 2). A light to moderate diffuse lymphoplasmocitary infiltrate was observed with the presence of macrophages, marked proliferation of fibroblasts and interstitial fibroplasia (Fig. 3c, bull 2). Scarce to moderate lymphoplasmacytic infiltrates and moderate to intense fibroplasia were observed in the pampiniform plexus. An intense inflammatory infiltrate was also observed in the testicular parenchyma accompanied by different degrees of germline atrophy in the seminiferous tubules with disappearance of the various layers of spermatids to spermatocytes (various degrees of testicular degeneration) in bulls 3 and 7 (Fig. 4b). In four bulls (1, 2, 4 and 6), the germinal epithelium completely disappeared, leaving only the Sertoli cells (testicular degeneration with Sertoli cell-only syndrome) (Fig. 4a). Desquamation of aberrant spermatids with multinucleated appearance and condensed chromatin were also observed in bull 3, characteristic of an alteration in the development of the germ line. Testicular parenchyma of the remaining bull 5 was autolytic, and lesions were not studied.

## Discussion

The effect of acute besnoitiosis has been studied for the first time in seven *B. besnoiti* naturally infected breeding bulls. We have demonstrated that a variety of severe acute lesions impair fertility in an early stage of the disease, as evidenced by different degrees of germline atrophy in the seminiferous tubules that are characteristic of an alteration in the development of the germ line and in a final step of testicular degeneration. Herein, a combination of serological, molecular and histopathological techniques was employed to characterize these case reports.

All the sampled bulls showed clinical signs and/or macroscopic lesions compatible with acute bovine besnoitiosis mainly characterized by fever, orchitis and hydrocele. The laboratory diagnostic approach followed confirmed acute besnoitiosis in all bulls. However, the results showed that the infection progress differed among the sampled animals. Four bulls showed high IgM values in the absence of IgG (bulls 4, 5, 6 and 7). These results confirmed an acute infection within the first 2–3 weeks post-infection and prior to IgG seroconversion. Diezma-Díaz et al. [19] showed that animals develop noticeable IgM levels from 7 days pi and remain high until 3 weeks pi. Afterwards, IgM levels begin to decrease and may remain detectable for a few years in chronically infected cattle. In contrast, both IgM and IgG antibodies were detected in bull 1, indicating an older infection than in the other bulls. However, the existence of a recent infection in this bull was corroborated by the low AI values obtained [19, 24]. Diezma-Diez et al. [19] described low avidity values coexisting with visible tissue cysts from 49 days post-infection onwards, and Schares et al. [24] reported that IgG avidity maturation is slower than cyst development.

The presence of small tissue cysts (25–90 μm) in three bulls (1, 2 and 6) also suggested a recent infection. Tissue cysts with a diameter between 48–67 μm were named juvenile tissue cysts by Basson et al. [12] since they have not reached the size of a fully developed mature cyst (> 300 μm). This result agrees with the serological results. Three out of four IgG seronegative bulls (4, 5 and 7) did not develop tissue cysts, whereas the remaining bull (bull 6) presented tissue cysts (mean diameter of 25 µm) compatible with an infection at 11–15 days post-infection [12]. In contrast, juvenile tissue cysts with a mean size of 50 µm were present in tissues from bull 1 that had already developed specific IgG. This tissue cyst size may be compatible with a 20-day post-infection period [12]. The structure of the juvenile cyst is consistent with the initial description made by Basson et al. [12] and more recently by Langenmayer et al. [13]. The cysts had a homogeneous, acellular and slightly basophilic external capsule and between 5–20 rounded cell nuclei, and a small number of bradyzoites were visualized inside them. When the PAS stain was used, the content of the cyst was observed since the amylopectin granules are stained, which are very abundant in the bradyzoite stage [9].

The parasite was able to spread through the organism, multiply and infect the target tissues of reproductive tract. McCully et al. [10] postulated that tissue cysts frequently appear in the same tissues where the initial proliferation of the parasite occurred with preference for small vessels. In our study, tissue cysts were observed in vascularized areas. In particular, scrotal skin cysts were mostly found in the papillary layer, a very vascularized and loose tissue, compared to the reticular stratum, where a fewer number of tissue cysts were present in agreement with previous observations [10, 13]. In four bulls (3, 4, 5 and 7), tissue cysts were not detected, but in bull 6, the presence of tachyzoites was suspected by immunohistochemistry. In particular, the presence of particulate antigen was detected, and a low *B. besnoiti* DNA burden was present in these tissues. This low parasitic load may be due to the initial replication of the tachyzoite in four bulls (3, 4, 5 and 7) and the detection of small cysts with a low number of bradyzoites inside [12] in the remaining bulls (1, 2 and 6). It has been described that a single parasite contains approximately 0.01 pg of *B. besnoiti* DNA [30]. However, studies carried out during the chronic phase detected a load of 10^7^–10^10^ parasites/25 g of tissue (4^8^–4^11^ zoites/mg of tissue) [26, 31], where mature cysts were abundant and reached a size of up to 400 μm with 200,000 bradyzoites [32]. The tachyzoite stage is predominantly responsible for lesions characteristic of endothelial damage and has previously been described in skin blood vessels [12]. In fact, bulls 3, 4 and 7 without cysts that were PCR positive showed characteristic lesions of acute infection coexisting with characteristic lesions of chronic besnoitiosis. However, the presence of cysts might aggravate certain lesions, such as thrombosis.

In our study, similar lesions were detected in the three testicular tissues analysed. A coexistence of lesions compatible with acute and chronic has been previously described in a naturally infected bull [9] and experimentally infected cows [13]. These histological findings prove a rapid progression of the disease. Tachyzoites might induce endothelial damage in small blood vessels followed by an inflammatory infiltrate that ends up with a marked proliferation of fibroblasts and interstitial fibroplasia in the testicular parenchyma (moderate diffuse lymphoplasmacytic sclerosing type orchitis), along with hyperkeratosis, ectasias of the sweat glands, acanthosis and angiogenesis.

**Fig. 5:**
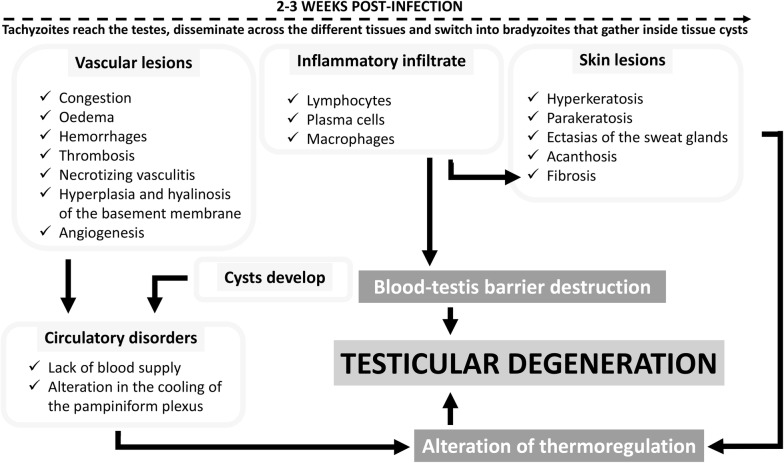
Pathogenesis of acute bovine besnoitiosis in the testicles. Proposed sequence of events according to the present histopathological findings

We hypothesized that endothelial injury and inflammation are key pathogenic mechanisms responsible for testicular degeneration in acutely infected bulls (Fig. 5). To the best of our knowledge, this study is the first report where testicular degeneration has been described in bulls with acute besnoitiosis, demonstrating the impact of acute besnoitiosis infection in bull fertility. This testicular degeneration might be a consequence of (i) thermoregulation failure induced by the tachyzoite stage and vascular lesions; (ii) severe vascular wall injury induced by the inflammatory response in the testis; and (iii) blood-testis barrier damage induced by a T cell response with increased production of pro-inflammatory cytokines and immune response. The alteration in thermoregulation might play a key role and would initially occur as a consequence of the vascular lesions [33] and, later, by the fibroplasia, hyperkeratosis, acanthosis and sweat glands ectasias [3]. Vascular lesions can cause a lack of irrigation, hindering the cooling of the testicle at the level of the pampiniform plexus aggravated by the presence of tissue cysts that may occlude the lumen of the vessel [33]. On the other hand, fibroplasia, hyperkeratosis and ectasias of sweat glands would hinder the cooling of the testicle [34]. During chronic besnoitiosis, there is a lack of heat exchange in the testicle due to thickening of the scrotum, which may contribute to permanent infertility [35]. In addition, cysts present in the tunica dartos, vaginal and albuginea may contribute to alter testicle cooling. According to the observations of Schulz et al. [6], fibrosis occurs, and inflammatory cells can be detected together with an interstitial lymphoplasmacytic orchitis associated with the presence of cysts [9]. In fact, Kumi-Diaka et al. [14] observed similar lesions and alterations in spermatogenesis in naturally and chronically infected bulls. Finally, the intense inflammatory infiltrate observed in the testicular parenchyma characteristic of orchitis might alter the haemato-testicular barrier by exposure of the sperm antigens to the immune system. In this scenario, the components of the luminal compartment (spermatocytes and spermatids) could be altered by an immune response, and the basal compartment (Sertoli cells and spermatogonia) may remain unaffected, as occurred in this study. However, this hypothesis requires further investigation. Moreover, whether bulls with oligospermia end up with testicular degeneration remains a possibility since the total duration of spermatogenesis in bulls is 61 days, and the duration of the seminiferous epithelium cycle is 13.5 days [36].

## Conclusions

This study confirmed that severe acute besnoitiosis leads to early sterility that might be permanently supported by the severe lesions observed. The tachyzoite stage would trigger a cascade of lesions which worsen due to tissue cyst development. A rapid progression of the disease has been shown by the coexistence of characteristic lesions of both acute (vascular injury and inflammation) and chronic besnoitiosis (fibrosis and hyperkeratosis of the skin) that ends up with an alteration in testicle thermoregulation and the blood-testis barrier. However, markers of disease progression and prognosis remain to be clarified.

## Data Availability

Data supporting the conclusions of this article are included within the article.

## Abbreviations

ELISA: enzyme-linked immunosorbent assay
WB: western blot
IgG: immunoglobulin G
IgM: immunoglobulin M
HE: haematoxylin and eosin
PAS: periodic acid-Schifff
PCR: polymerase chain reaction
qPCR: quantitative real-time PCR
